# Radical Pancreaticoduodenectomy for Benign Disease

**DOI:** 10.1100/tsw.2008.147

**Published:** 2008-11-22

**Authors:** D. O. Kavanagh, C. O'Riain, P. F. Ridgway, P. Neary, T. C. Crotty, J. G. Geoghegan, O. Traynor

**Affiliations:** ^1^ Liver Unit, St. Vincent's University Hospital, Elm Park, Dublin, Ireland; ^2^ Department of Pathology, St. Vincent's University Hospital, Elm Park, Dublin, Ireland

**Keywords:** pancreaticoduodenectomy, endoscopic ultrasound, chronic pancreatitis, choledochal cyst, Whipple's

## Abstract

Whipple's procedure is the treatment of choice for pancreatic and periampullary malignancies. Preoperative histological confirmation of malignancy is frequently unavailable and some patients will subsequently be found to have benign disease. Here, we review our experience with Whipple's procedure for patients ultimately proven to have benign disease. The medical records of all patients who underwent Whipple's procedure during a 15-year period (1987–2002) were reviewed; 112 patients underwent the procedure for suspected malignancy. In eight cases, the final histology was benign (7.1%). One additional patient was known to have benign disease at resection. The mean age was 50 years (range: 30–75). The major presenting features included jaundice (five), pain (two), gastric outlet obstruction (one), and recurrent gastrointestinal haemorrhage (one). Investigations included ultrasound (eight), computerised tomography (eight), endoscopic retrograde cholangiopancreatography (seven; of these, four patients had a stent inserted and three patients had sampling for cytology), and endoscopic ultrasound (two). The pathological diagnosis included benign biliary stricture (two), chronic pancreatitis (two), choledochal cyst (one), inflammatory pseudotumour (one), cystic duodenal wall dysplasia (one), duodenal angiodysplasia (one), and granular cell neoplasm (one). There was no operative mortality. Morbidity included intra-abdominal collection (one), anastomotic leak (one), liver abscess (one), and myocardial infarction (one). All patients remain alive and well at mean follow-up of 41 months. Despite recent advances in diagnostic imaging, 8% of the patients undergoing Whipple's procedure had benign disease. A range of unusual pathological entities can mimic malignancy. Accurate preoperative histological diagnosis may have allowed a less radical operation to be performed. Endoscopic ultrasound–guided fine needle aspirate (EUS-FNA) may reduce the need for Whipple's operation in benign pancreaticobiliary disease in the future.

## INTRODUCTION

Currently, Whipple's procedure is the treatment of choice for suspected pancreatic head and periampullary malignancies. It has recognised perioperative mortality rates of 1–5% and morbidity rates of approximately 40%[1,2,3]. Despite this, it offers the only potential cure for pancreatic adenocarcinoma, which is currently the fourth leading cause of cancer-related mortality in Europe[4].

In the past, confirming a preoperative histological diagnosis was almost essential in the management of patients with suspected pancreaticobiliary malignancies. Advances in imaging have improved the ability to make a preoperative radiological diagnosis. This, coupled with a reduction of the morbidity and mortality of Whipple's procedure, has resulted in less emphasis on preoperative tissue diagnosis. Despite these advances, a number of patients who undergo Whipple's procedure for suspected malignancy are subsequently found to have benign disease. The major series in the literature of Whipple's procedure performed for presumed malignancy report that 5–10% of patients ultimately have benign disease proven[4,5,6,7].

In 1995, Yoshida et al.[8] first described a rare variant of chronic pancreatitis called autoimmune pancreatitis. This condition typically affects elderly Asian men and presents with obstructive jaundice. Pathognomic radiological findings include a "sausage-shaped" enlarged pancreas on CT imaging. Elevated serum IgG4 is often found. It responds to corticosteroid therapy.

Certain circumstances do warrant increased attempts to make a preoperative tissue diagnosis. These include patients suitable for neoadjuvant therapy or palliation based on imaging, and in cases where there is diagnostic doubt, such as lymphoma, TB, benign strictures, and focal pancreatitis. In these circumstances, we utilise an algorithm as illustrated in Table 1. Techniques to acquire a preoperative tissue diagnosis prior to pancreatic resection are limited by a poor diagnostic yield. Percutaneous image-guided fine-needle aspirates for cytology (FNA) have reported sensitivity rates of 70–90%. However, the potential for needle tract seeding (1.2%) and transcoelomic spread remains to be fully elucidated. Its role is further limited by the possibility of sampling error. Endoscopic retrograde cholangiopancreatography (ERCP) biopsy and brushings, or transpapillary biopsy, have reported sensitivities of 30–60%[9,10].

Intraoperative Tru-cut biopsy and frozen section is limited by sampling error and difficulty in distinguishing between chronic pancreatitis and pancreatic cancer. In our institution, we have observed a spectrum of benign pathological entities that mimic pancreatic and periampullary malignancies. The patients did not have a preoperative histological diagnosis and underwent Whipple's procedure. The more widespread availability of endoscopic ultrasound (EUS) in combination with FNA and improvements in other diagnostic modalities is likely to improve the diagnostic accuracy and reduce the need for radical resections in benign disease[11].

We reviewed the clinical, radiological, and pathological characteristics of patients in our unit who underwent Whipple's procedure for suspected, but unproven, malignancy.

## MATERIALS AND METHODS

The medical records of all patients who underwent Whipple's procedure from January 1, 1987 to December 31, 2002 at The Liver Unit, St Vincent's University Hospital, were reviewed. Data were acquired from multiple sources, including the Hospital Inpatient Enquiry System (HIPE), operative records, and radiological databases. Information on presenting symptoms, preoperative radiological and histological workup, operative findings, morbidity, mortality, and pathological findings was obtained from the records of patients with benign pathology. Patients were followed up for a minimum of 12 months (range: 12–120).

**TABLE 1**
**Current Algorithm to Evaluate a Patient with Suspected Pancreaticobiliary Malignancy**

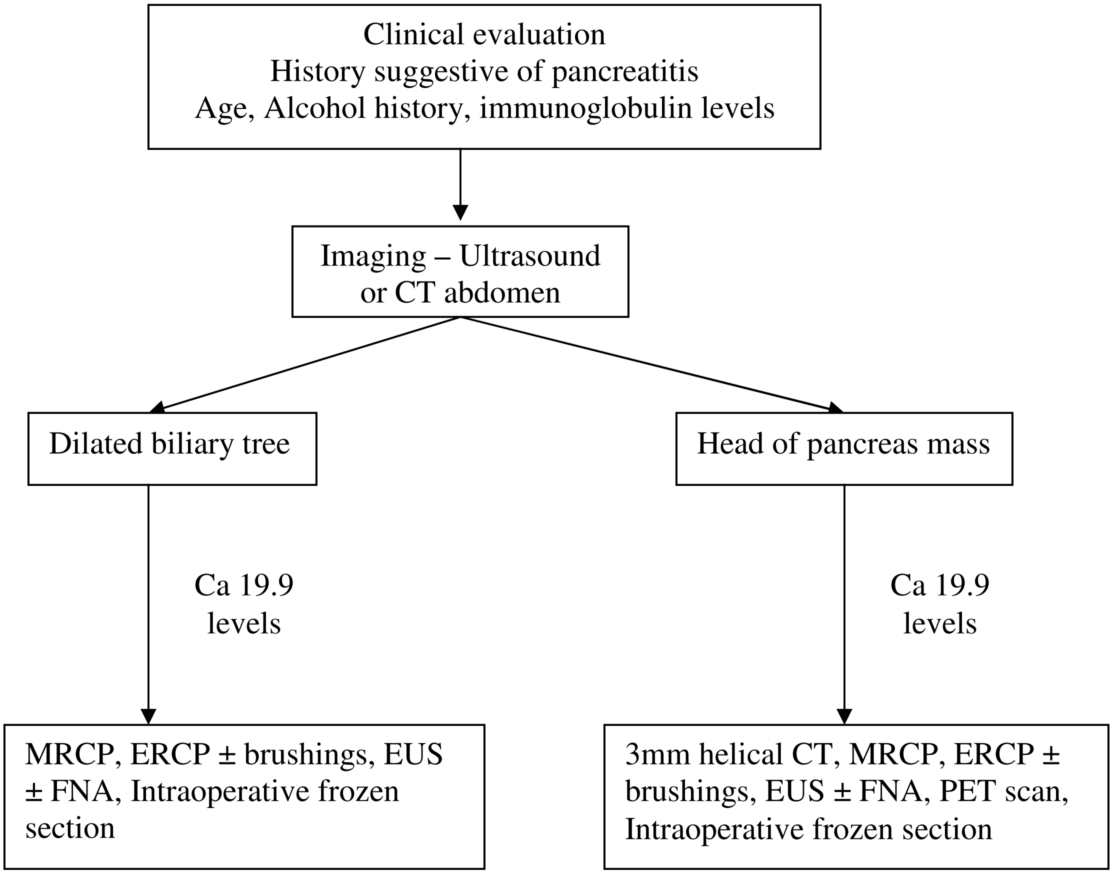

## RESULTS

A total of 112 patients underwent radical pancreaticoduodenectomy for presumed malignant disease based on clinical, radiological, and cytological data. Of these, eight patients were subsequently found to have a variety of benign pathological diagnoses confirmed as shown in Table 2. An additional patient underwent radical pancreaticoduodenectomy for recurrent gastrointestinal haemorrhage, which was known to be due to benign duodenal angiodysplasia at the time of resection.

## Patient 1

A previously healthy 43-year-old man presented with painless obstructive jaundice and weight loss. He had no previous history of gastrointestinal symptoms and did not consume alcohol. Following initial clinical evaluation, he underwent abdominal US and CT, which demonstrated dilated intrahepatic and extrahepatic ducts down to the ampullary area (Figs. 1 and 2). There was no calcification seen within the pancreas and no radiological features of autoimmune pancreatitis. His Ca 19.9 level was 5 iu/L. The common bile duct (CBD) measured 12 mm in diameter. ERCP identified tapering of the distal CBD and a stent was passed.

**TABLE 2**
**Clinicopathological Features and Potential Procedure with Preoperative Tissue Diagnosis**

| No. | Clinical Features | Radiological Findings | Pathology | Potential Operation with EUS-FNA |
|---|---|---|---|---|
| 1 | Jaundice | Periampullary mass | Chronic pancreatitis with regional lymphadenitis | Biliary bypass |
| 2 | Pancreatitis | Pancreatic duct stricture | Focal pancreatitis | Peustow procedure |
| 3 | Jaundice, weight loss | Pancreatic head mass | Inflammatory pseudotumour | Biliary bypass |
| 4 | Ascending cholangitis | CBD stricture | CBD stricture with focal sclerosing cholangitis | Biliary bypass |
| 5 | Ascending cholangitis | CBD stricture | CBD stricture with cholelithiasis | Biliary bypass |
| 6 | Pain | CBD stricture | Choledochal cyst with adjacent chronic cholangitis and pancreatitis | Cyst excision |
| 7 | Gastric outlet obstruction | Duodenal ulcerating mass | Cystic duodenal wall dysplasia | Gastrojejunostomy |
| 8 | Jaundice | CBD stricture | Granular cell tumour | Biliary bypass |

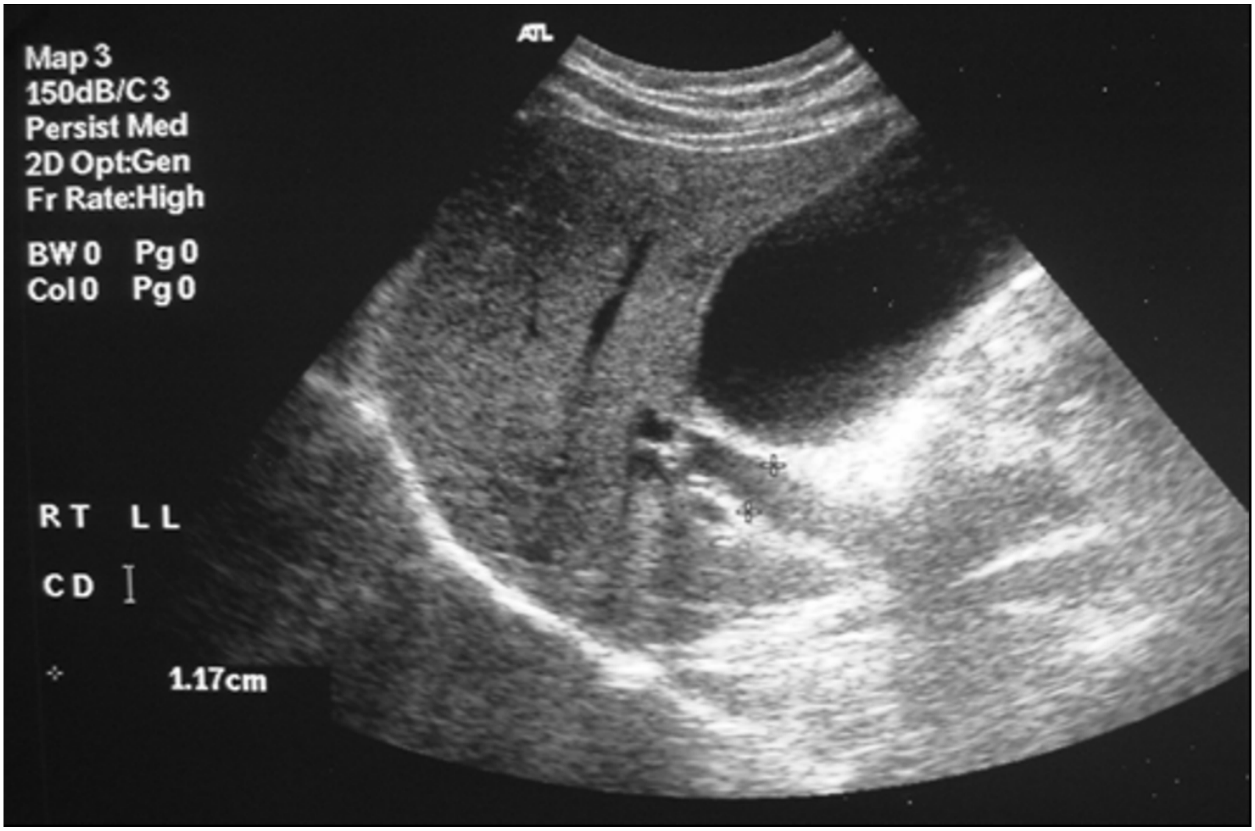

**FIGURE 1.** US demonstrates dilated intrahepatic and extrahepatic ducts, and CBD down to the lower end. The diameter of CBD is 1.17 cm.

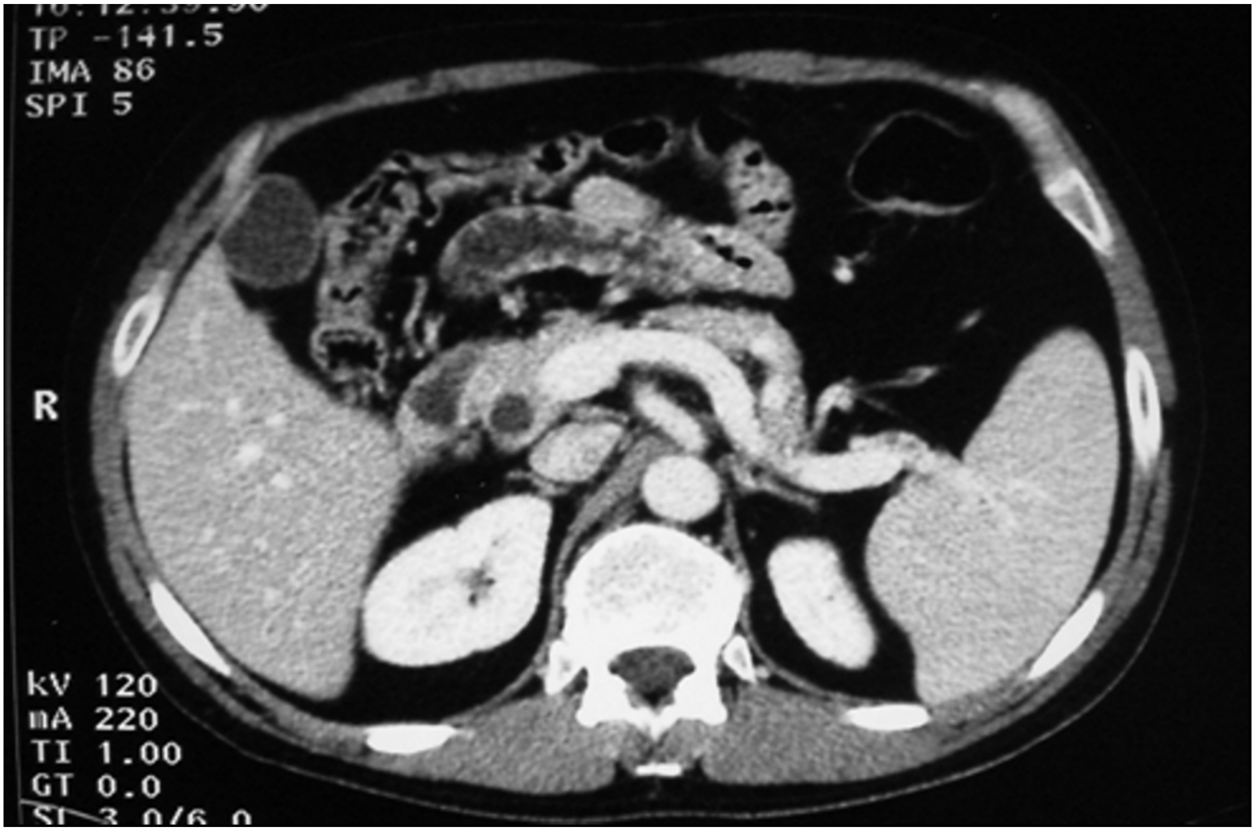

**FIGURE 2.** CT reveals a dilated CBD down to the level of the pancreatic head. No focal mass is seen.

EUS revealed a periampullary mass (Fig. 3). At the time, FNA was not available[11]. At operation, a mass was found in the head of the pancreas and a Whipple's procedure was performed. Histological examination revealed chronic pancreatitis with regional lymphadenitis. There was no evidence of lymphocytic infiltration. His postoperative recovery was complicated by a hepatic abscess, which required percutaneous catheter drainage.

## Patient 2

A 33-year-old female presented with acute pancreatitis in 1993. US and CT confirmed a dilated pancreatic duct with a mass in the head of the pancreas. A low-density capsular rim was not evident. ERCP was unsuccessful. At US-guided percutaneous pancreatogram, a stricture was identified proximally in the pancreatic duct. Cytological analysis of fluid aspirated revealed no malignant cells. Serum IgG4 was not available in this institution at the time of treatment. A Whipple's procedure was performed and histological evaluation demonstrated chronic focal pancreatitis with no lymphocytic or plasma cell infiltration.

## Patient 3

A 74-year-old male presented in 2000 with persistent painless obstructive jaundice, weight loss, and anorexia. CT confirmed a pancreatic mass. ERCP revealed a tight stricture in the CBD and a stent was inserted. Serum IgG4 levels were not available at that time. Brushings did not contain malignant cells. At

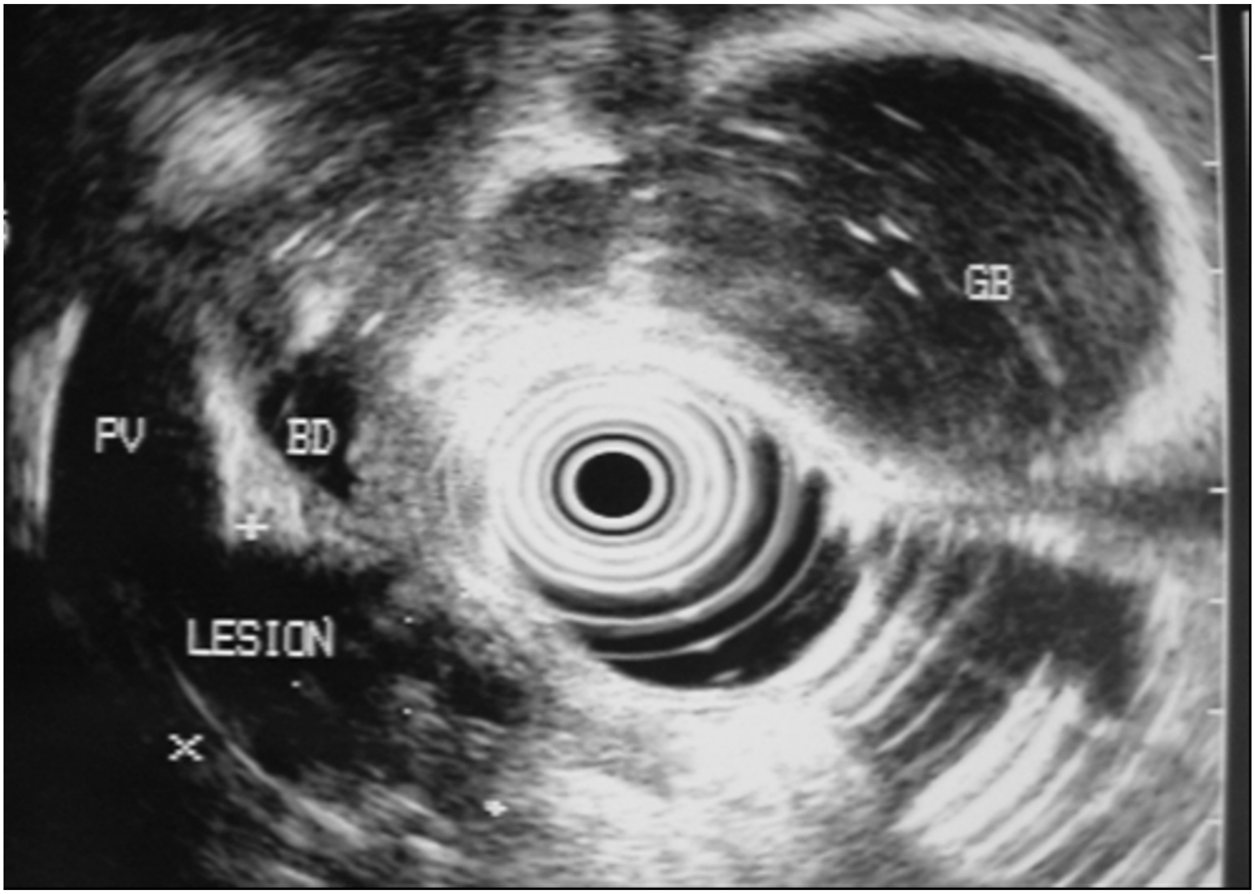

**FIGURE 3.** EUS shows an irregular periampullary mass identified in close proximity to the portal vein.

laparotomy, an irregular bulky mass in the pancreatic head was identified and a Whipple's procedure was performed for what proved to be an inflammatory pseudotumour. This contained spindle cells that were vimentin positive, but negative for muscle markers and abundant lymphocytes. He had a postoperative myocardial infarction from which he made a favourable recovery.

## Patient 4

A 62-year-old previously well female presented with ascending cholangitis in 1998. US and CT confirmed a dilated CBD (diameter: 15 mm), which abruptly narrowed at the level of the head of the pancreas. ERCP confirmed a tight stricture at this point and brushings were taken, which were negative for malignant cells. A stent was deployed. At laparotomy, a mass was identified in the head of the pancreas. Intraoperative needle biopsy for frozen section was negative for malignancy. A Whipple's procedure was performed. Histology confirmed a benign biliary stricture secondary to focal sclerosing cholangitis.

## Patient 5

A 61-year-old male presented with ascending cholangitis. Following initial antimicrobial therapy, he underwent a CT scan, which confirmed a mass in the head of the pancreas. ERCP revealed a stricture in the lower end of the CBD. A stent was inserted and brush cytology was negative for malignant cells. At laparotomy, a mass in the head of the pancreas and lower CBD was found, and he underwent resection. He developed an intra-abdominal collection, which required percutaneous drainage and insertion of a drainage catheter. Histological evaluation demonstrated a CBD stricture and cholelithiasis.

## Patient 6

A 63-year-old female presented in 1999 with persistent right upper quadrant pain with associated weight loss. US confirmed a dilated CBD (diameter: 11 mm) and CT identified a 4-cm mass in the head of the pancreas with a dilated biliary tree. Ca 19.9 was 11 iu/L. ERCP confirmed an irregular stricture at the distal end of the CBD. At laparotomy, choledochoscopy identified an apparent tumour at the lower CBD and a radical pancreaticoduodenectomy was performed. Histology demonstrated a 2.5-cm choledochal cyst with associated chronic cholangitis and pancreatitis.

## Patient 7

A 44-year-old male presented with persistent abdominal pain, nausea, vomiting, and weight loss. CT showed gastric outlet obstruction. EUS revealed an ulcerating duodenal mass. At laparotomy, a large diffuse pancreatic mass was observed, invading the duodenal wall and encroaching on the ampulla. He underwent a Whipple's procedure and required further laparotomy on the sixth postoperative day for an anastomotic leak. This was sutured and he made a complete recovery. Histology revealed the unusual entity, cystic duodenal wall dysplasia.

## Patient 8

A 30-year-old female presented in 1994 with painful obstructive jaundice. CT confirmed a mass in the head of the pancreas with CBD dilatation down to the level of the pancreas. ERCP showed a tapering stricture and a stent was deployed. At laparotomy, several needle biopsies for frozen sections were taken from the suspicious mass in the head of the pancreas, all of which were negative. Choledochoscopy revealed a tumour and a Whipple's procedure was performed. Histology revealed a granular cell tumour.

## Patient 9

This 43-year-old female was referred with a diagnosis of duodenal angiodysplasia. Over the previous 5 years, she required multiple hospital admissions for acute gastrointestinal haemorrhage. Despite multiple therapeutic interventions, including endoscopic photocoagulation and embolisation of the gastroduodenal artery, she continued to experience intermittent significant gastrointestinal haemorrhage. She underwent a Whipple's procedure for known benign disease and has not required further transfusion at 4-year follow-up.

None of the patients with benign disease had elevated Ca 19.9 assays prior to surgery. Fifty-five patients (53%) of the malignant cohort had elevated Ca 19.9. There were no mortalities in this series and the mean inpatient hospital stay was 23 days (range: 14–51). All patients remain alive and well with a mean follow-up of 64 months (range: 12–120).

## DISCUSSION

Pancreatic and periampullary carcinomas are devastating malignancies with reported 5-year survival rates of less than 25%[12]. Whipple's procedure offers the only hope of cure. In specialised centres, reported perioperative mortalities range from 1 to 5%[1,2]. The morbidity of this operation, both perioperatively and in the long term, is significant[3]. Improved availability of a variety of applicable diagnostic modalities, including high resolution CT, magnetic resonance imaging (MRI), and positron emission tomography (PET), has not been mirrored by advances in the area of preoperative tissue diagnosis[9,10]. As a result, Whipple's procedure is frequently performed in patients who do not have a preoperative histological diagnosis[13,14]. The rate of preoperative tissue diagnosis in the current series was 3%. Therefore, each series of Whipple's procedures inevitably includes a subset of patients with benign disease simulating malignancy based on clinical, radiological, and cytological data[4,5,6,7].

Herein, we report eight (7%) patients in a series of 112 who underwent Whipple's procedure for presumed malignant disease. An additional patient underwent surgery for known benign disease. All patients had clinical, radiological, endoscopic, and intraoperative findings consistent with malignancy.

US has reported sensitivity rates of 85% in detecting pancreatic malignancies[15,16]. However, in patients with chronic pancreatitis, up to 40% have a focal mass indistinguishable from malignancy[17]. US-guided FNA has a sensitivity of 84% and a specificity of 100% in evaluating suspicious lesions of the pancreatic head[18]. However, this technique is subject to sampling error and the smaller lesions that are more likely to be resectable are more likely to be missed. None of our patients had masses detectable on US.

CT has a reported sensitivity of greater than 90% in detecting pancreatic malignancies and a specificity of 50%. Differentiating between malignancy and chronic pancreatitis is not always possible and in such cases, clinical characteristics, such as a history of alcohol excess, may have a major role in determining the diagnosis[19,20]. CT-guided sampling is limited by sampling error and sensitivity rates rarely exceed 70%[21]. Furthermore, there is a small, but recognised, risk of serious complications[22].

MRCP is a noninvasive modality with a sensitivity of up to 95% and specificity of 97%. Hänninen et al. report four of 17 confirmed cases of chronic pancreatitis misdiagnosed as malignancy with MRCP[23].

ERCP is frequently used as both a diagnostic and therapeutic modality in pancreaticobiliary neoplasms. Diagnostic sampling can be performed by aspiration of bile or pancreatic juice, brush cytology, intraductal FNA, and transpapillary biopsy. Several authors have reported disappointing results with brush cytology with sensitivities ranging from 50 to 70%. The diagnostic yield can be improved to 77% (when atypia is considered malignant) by increasing the number of tissue sampling techniques used[24]. Technical modifications, such as the use of a 10-Ch dilator attached to a Velcro pad, have reported 100% sensitivity rates in a series of 15 patients. This result has not been reproduced by any other author[25]. While a positive result confirms the diagnosis, the problem of false-negatives is significant. There is also significant morbidity with an 11% rate of acute pancreatitis reported in some series[26].

PET using 2-[18-F] fluoro-2-deoxy-D-glucose (FDG) takes advantage of the metabolic differences between benign and malignant disease. The European consensus has designated differentiation of benign from malignant pancreatic disease as an indication for its use[27]. Sensitivity and specificity rates of approximately 85 and 88%, respectively, have been reported[28,29]. In a study of 106 patients with pancreatic masses (74 with pancreatic cancer and 32 with chronic pancreatitis), the overall sensitivity and specificity were 85 and 84%, respectively. False-positives occurred in inflammation, while false-negatives occurred in patients with elevated glucose levels[30]. The recent development of software, which facilitates digital image fusion of CT and PET, has increased the sensitivity to 90%, while the specificity remains unchanged[31].

Image-guided FNA rarely reports sensitivity rates greater than 90%[18]. The potential for seeding of malignant cells is a cause for concern. Implantation of the tumour along the percutaneous tract has been reported, but is considered a rare complication (0.001%)[32]. The potential for transcoelomic seeding is more significant. Warshaw[33] reported on 40 patients with pancreatic cancer deemed respectable on imaging. One-third (13) had malignant cells in a peritoneal aspirate. In those who underwent previous

FNA, 75% (6/8) had positive cytology. In patients who did not undergo cytological analysis, 19% (6/32) had a positive cytology. He proposed that FNA converted resectable disease into disseminated malignancy[33,34]. His findings were supported by Nakatsuka et al.[35]and Yachida et al.[36], who also showed that positive peritoneal cytology is associated with poorly differentiated pancreatic cancer with a significantly worse survival. The MD Anderson group found a much lower incidence (7%) of positive peritoneal cytology despite undergoing percutaneous FNA[37].

The development of adjuvant and neoadjuvant therapies in pancreatic cancer, and the need to confirm the diagnosis prior to treatment has revived the emphasis on tissue diagnosis. The potential for longer survival means the issue of peritoneal seeding is more relevant than when the survival was <18 months[38,39,40].

The introduction of EUS-guided FNA (EUS-FNA) overcomes the aforementioned limitations and may allow the diagnosis to be established without exploratory surgery. Its ability to be in close proximity to target lesions eliminates artefact due to overlying bowel gas, while its ability to deliver high frequency waves generates improved resolution of images and associated lymphovascular structures. It is as good as CT in detecting lesions >3 cm. Its main role is in the evaluation of lesions <3 cm that may be missed on CT[41,42,43,44]. Chang et al. reported on 44 patients who underwent EUS-FNA of pancreatic lesions and associated lymph nodes. They reported a sensitivity of 92%, specificity of 100%, and diagnostic accuracy of 95% for pancreatic lesions. This impacted positively on patient care, and avoided surgical exploration in 27% and further investigation in 57% of patients[45,46]. In established units, sensitivity rates of 80–90% have been reported[47]. Chandrajit et al. described their experience with EUS-FNA of the pancreas. EUS-FNA failed to identify any malignant cells in 14 of the 15 patients subsequently proven to have benign disease. Furthermore, their false-negative rate of 7% is similar to that of other modalities without any increase in morbidity[48]. EUS-FNA is safe and reliable. It may eliminate the risk of peritoneal seeding, while facilitating more accurate needle placement. It can distinguish focal pancreatitis from carcinoma. However, it requires skilled endoscopists with extensive knowledge of pancreatic sonographic anatomy. The inherent risk of a false-negative in a small resectable tumour often prompts resection as the surgeon may err on the side of caution[49].

All of our patients had significant symptomatology and radiological findings at presentation. In view of the considerable diagnostic limitations associated with current imaging modalities, a preoperative histological diagnosis is not always accurate. Each patient in this series required some intervention. The magnitude of the intervention may have been reduced if malignancy could have been excluded with confidence preoperatively as illustrated in Table 2. Pancreatic cancer has a 5-year overall survival of less than 4%. Whipple's procedure is the only treatment with a curative potential. This was performed in each patient in our series. There was no operative mortality in our group. Whipple's procedure has recognised perioperative mortality rates of 0–5%[1,2,3,50]. Morbidity included intra-abdominal collection (one) and a liver abscess (one). These required percutaneous catheter drainage. An anastomotic leak occurred, which required laparotomy and suturing. An additional patient developed a myocardial infarction. The mean inpatient hospital stay was 23 days (range: 14–51). The major series report morbidity rates of approximately 40%[1,2,3,50]. These may include anastomotic leakage (5–7%), intra-abdominal bleeding (3–6%), intra-abdominal abscess (5–10%), and relaparotomy (3.7–9%)[51,52].

The current series includes a variety of unusual pathological entities masquerading as pancreatic cancer. Although granular cell tumours can arise from any part of the body, granular cell tumour of the pancreas is rare. They are usually benign, however, complete excision is needed as malignancy has been reported[53]. Inflammatory pseudotumours are benign, rare, tumour-like lesions of uncertain pathogenesis. These can occasionally have radiographic features of malignancy, as in our case[54]. Choledochal cysts are usually diagnosed in the first few years of life. Presentation in adulthood is uncommon and often associated with complications of the cyst. These include anastomotic stricture, cholangitis, biliary calculi, and biliary tract malignancy[55]. In the current series, the resected specimen in a single case contained a 2.5-cm choledochal cyst with associated chronic cholangitis. This had radiographic and endoscopic features of malignancy at the lower end of the CBD. Whipple's procedure for cystic duodenal wall dysplasia mimicking malignancy has not been previously described in the medical literature. The remaining resected specimens with malignant features included focal pancreatitis (two) and CBD strictures (two).

The recognition of the entity, autoimmune pancreatitis, and its effective treatment with steroid therapy may reduce the need for resection for presumed malignancies later found to be benign. None of our patients had autoimmune pancreatitis. However, widespread awareness of the pathognomic features of this condition was not available in the 1990s. The resected specimen of Patient 3 was labelled a benign pseudotumour, but there was an abundant lymphocytic infiltrate. Immunoglobulin markers were not available at the time[56]. His preoperative and operative findings were consistent with a pancreatic malignancy.

This series involves a heterogeneous group of patients with a variety of pathologies masquerading as malignancy over the last 15 years. The evolution of preoperative acquisition of a tissue diagnosis has been mirrored by significant improvement in imaging. The refinements of imaging provided by high-resolution CT and its more widespread availability generates case-specific information that may deem something previously locally invasive as now more likely to be benign. These advancements, coupled with reported sensitivity rates of >90% for EUS-guided cytology and the ability of PET to distinguish benign from malignant pancreatic lesions, may eliminate the need for radical resection in select cases where the overall risk of malignancy is extremely low based on the algorithm as demonstrated in Table 1.

## CONCLUSION

Over the past few decades, there has been a shift in the approach of the surgical oncologist towards the management of pancreaticobiliary malignancy. In the past, surgeons were reluctant to perform surgery without a preoperative tissue diagnosis. Advances in imaging and poor diagnostic yield with brush cytology, and the concerns about tumour dissemination with FNA, resulted in a change in management with surgeons performing resections without a tissue diagnosis. This may revert to the traditional approach with the advent of EUS-FNA. A variety of unusual benign pathological entities can mimic pancreaticobiliary malignancies. Improvements in diagnostics, awareness of autoimmune pancreatitis, and accurate preoperative histological diagnosis may have avoided resection or allowed a less radical operation to be performed. Increasing availability of EUS-FNA may reduce the need for Whipple's operation in benign disease in the future.
